# Roflumilast partially reverses smoke-induced mucociliary dysfunction

**DOI:** 10.1186/s12931-015-0294-3

**Published:** 2015-10-31

**Authors:** Andreas Schmid, Nathalie Baumlin, Pedro Ivonnet, John S. Dennis, Michael Campos, Stefanie Krick, Matthias Salathe

**Affiliations:** Division of Pulmonary, Allergy, Critical Care and Sleep Medicine, University of Miami Miller School of Medicine, 1600 NW 10th Ave, RMSB #7058, Miami, FL 33136 USA

**Keywords:** Roflumilast, FRET, Cilia, Airways, CFTR, Mucociliary clearance

## Abstract

**Background:**

Phosphodiesterases (PDEs) break down cAMP, thereby regulating intracellular cAMP concentrations and diffusion. Since PDE4 predominates in airway epithelial cells, PDE4 inhibitors can stimulate Cystic Fibrosis Transmembrane Conductance Regulator (CFTR) by increasing cAMP. Tobacco smoking and COPD are associated with decreased CFTR function and impaired mucociliary clearance (MCC). However, the effects of the PDE4 inhibitor roflumilast on smoke-induced mucociliary dysfunction have not been fully explored.

**Methods:**

Primary normal human bronchial epithelial cells (NHBE) from non-smokers, cultured at the air-liquid interface (ALI) were used for most experiments. Cultures were exposed to cigarette smoke in a Vitrocell VC-10 smoking robot. To evaluate the effect of roflumilast on intracellular cAMP concentrations, fluorescence resonance energy transfer (FRET) between CFP- and YFP-tagged protein kinase A (PKA) subunits was recorded. Airway surface liquid (ASL) was measured using light refraction scanning and ciliary beat frequency (CBF) employing infrared differential interference contrast microscopy. Chloride conductance was measured in Ussing chambers and CFTR expression was quantified with qPCR.

**Results:**

While treatment with 100 nM roflumilast had little effect alone, it increased intracellular cAMP upon stimulation with forskolin and albuterol in cultures exposed to cigarette smoke and in control conditions. cAMP baselines were lower in smoke-exposed cells. Roflumilast prolonged cAMP increases in smoke-exposed and control cultures. Smoke-induced reduction in functional, albuterol-mediated chloride conductance through CFTR was improved by roflumilast. ASL volumes also increased in smoke-exposed cultures in the presence of roflumilast while it did not in its absence. Cigarette smoke exposure decreased CBF, an effect rescued with roflumilast, particularly when used together with the long-acting ß-mimetic formoterol. Roflumilast also enhanced forskolin-induced CBF stimulation in ASL volume supplemented smoked and control cells, confirming the direct stimulatory effect of rising cAMP on ciliary function. In active smokers, CFTR mRNA expression was increased compared to non-smokers and ex-smokers. Roflumilast also increased CFTR mRNA levels in cigarette-smoke exposed cell cultures.

**Conclusions:**

Our results show that roflumilast can rescue smoke-induced mucociliary dysfunction by reversing decreased CFTR activity, augmenting ASL volume, and stimulating CBF, the latter particularly in combination with formoterol. As expected, CFTR mRNA expression was not indicative of apical CFTR function.

## Background

The airways are continuously exposed to dust and infectious agents. To prevent damage caused by irritants, they require effective clearing mechanisms, the physiologically most important one being mucociliary clearance (MCC). Two major determinants of effective MCC are directly regulated by cAMP: Ciliary beat frequency (CBF) [1] and the Cystic Fibrosis Transmembrane Conductance Regulator (CFTR), a major anion channel regulating airway surface liquid (ASL) volume [2].

Phosphodiesterases (PDEs) belong to a family of enzymes that catalyze the breakdown of the second messengers cAMP and cGMP to their inactive forms [3]. PDE4 gene products have a higher affinity to cAMP (K_m_ 1–10 μM) than cGMP (K_m_ > 50 μM) and reveal a broad tissue distribution, including the brain, gastrointestinal tract, spleen, lung, heart, testis and kidney [4]. The lack of effective drug therapy for chronic obstructive pulmonary disease (COPD) has increased the interest in PDE4 inhibition in recent years [5], as PDEs are not only highly expressed in airway epithelial cells, but also in leukocytes and other inflammatory cells, which are involved in the pathogenesis of COPD [3]. Roflumilast, a PDE4 inhibitor with an IC_50_ of 0.6 nM [3], has been approved in the United States and Europe to reduce the risk of COPD exacerbations in patients with severe COPD associated with chronic bronchitis and a history of exacerbations [6].

CFTR function is decreased in patients with COPD [7, 8] and in smokers [9]. Furthermore, PDE inhibitors have been shown to increase chloride transport through CFTR [10]. PDE4 is one of the major isoforms expressed in the airways [11]. These observations suggest that PDE inhibitors could play a key role in improving MCC by restoring depleted ASL in patients with COPD as well as in smokers.

In this study, we examined the role of roflumilast on parameters of MCC in normal human bronchial epithelial (NHBE) cells. We found that roflumilast increased intracellular cAMP concentrations, improved CFTR function and enhanced CBF in cigarette smoke-exposed NHBE cells. For some of these parameters, formoterol had an additive effect. These results indicate that roflumilast rescues at least part of the dysfunctional MCC after acute smoke exposure.

## Methods

### Chemicals

Roflumilast was provided by Forest Laboratories Inc. (New York, NY, USA). All other chemicals were purchased from Sigma (St. Louis, MO, USA), unless otherwise specified.

### Cell cultures

Normal human bronchial epithelial (NHBE) cells from non-smokers were isolated from lungs unsuited for transplant and generously donated for research by organ donors. These lungs were recovered with IRB approved consents by the LifeCenter Northwest in Washington State and the Life Alliance Organ Recovery Agency of the University of Miami. After isolation as previously described [12–15], NHBE cells were dedifferentiated through expansion on plastic and redifferentiated at an air-liquid interface (ALI) on collagen-coated Transwell permeable supports (12 mm, 24 mm and Snapwell, Costar Corning, Tewksbury, MA, USA). NHBE cells were considered fully differentiated after 3–4 weeks at the ALI as assessed by beating cilia and mucus transport.

### Smoke exposure of NHBE ALI cultures

Fully differentiated NHBE cells were exposed to cigarette smoke using a Vitrocell VC-10 smoking robot (Vitrocell Systems GMBH, Waldkirch, Germany). Four cigarettes (University of Kentucky 3R4F) were smoked according to ISO standard 3308: six puffs per cigarette with a 35 mL volume per puff and a waiting time between each puff of 60 s. Roflumilast was added 1 h before and re-added after smoking. CBF, FRET, and CFTR conductance were measured 3 h after smoke exposure. After these measurements, NHBE cells were lysed and lysate stored at −20 °C for quantitative PCR.

### Pseudotyped Lentivirus Vectors and Infection of Airway Epithelial Cells

Third generation, propagation-deficient, HIV pseudotyped lentiviruses encoding fluorescently tagged PKA subunits (RII-CFP and CAT-YFP) under the transcriptional control of the ciliated cell-specific *foxj1* promoter were used for FRET as described previously [13]. Briefly, recombinant lentiviruses were constructed using the pRRLsinPPT.CMV.MCS.Wpre vector [16]. For the initial constructs, genes encoding the catalytic PKA subunit CAT and the regulatory PKA subunit RII, fused to the fluorescent proteins YFP and CFP, respectively [17], were cloned into the multiple cloning site downstream of the ciliated cell-specific *foxj1* promoter for sole expression in ciliated cells [18]. Using calcium phosphate co-precipitation (Clontech Laboratories, Inc., Mountain View, CA, USA), lentiviruses were prepared by co-transfecting HEK 293 T cells with vector and packaging DNAs plasmids. Virus-containing medium was collected 48 h and 72 h later, concentrated by polyethylene glycol (11 %) precipitation, and stored at −80 °C. An estimation of the virus titer was performed using the p24 HIV Antigen ELISA kit (PerkinElmer, Wellesley, MA, USA). Dedifferentiated cells were used for co-infection with both viral constructs. At the time of plating the cells on Transwells, virus was added at a ratio of 100 ng per 500,000 cells in bronchial epithelial growth medium (BEGM) containing polybrene (2 μg/ml final concentration). The infection was done overnight, at 37 °C in 5 % CO_2_. The following day, virus was removed, and BEGM was changed to ALI medium top and bottom until cells reached confluence, when an air liquid interface was created. Expression of the fluorescently tagged proteins was monitored using an inverted fluorescence microscope.

### Measurement of CBF and FRET in airway epithelial cells

Fully differentiated NHBE cells cultured on 24 mm Transwell supports were placed in a customized, fully enclosed chamber, allowing independent perfusion of the apical and basolateral compartments. The chamber was mounted at room temperature on the stage of an upright Nikon E600fn microscope. Water was added on top of the closed chamber for use of a 63× water immersion objective with a numerical aperture of 1.0. FRET was measured as described previously [13], with images acquired every 10s. CBF was recorded according to published methods [13, 19], using infrared differential interference contrast video microscopy. CBF and FRET were measured in real time and simultaneously in ciliated cells that expressed both fusion proteins. In addition, CBF was also recorded on an inverted Zeiss Axiovert without apical perfusion before and after apical DPBS supplementation.

### Ussing chamber experiments

Snapwell filters containing fully differentiated NHBE cells were rinsed with Krebs-Henseleit solution (KH), and then mounted in Ussing chambers (EasyMount Chambers; Physiologic Instruments, San Diego, CA, USA) containing KH in apical and basolateral chambers. Solutions were maintained at 37 °C by heated water jackets, and were continuously bubbled with a 95 % room air / 5 % CO_2_ mixture to maintain the pH at 7.4. To monitor short-circuit current (I_SC_), the transepithelial membrane potential was clamped at 0 mV with a six-channel voltage clamp (model VCC MC6; Physiologic Instruments) using Ag/AgCl electrodes in agar bridges. Signals were digitized and recorded with DAQplot software (VVI Software, College Station, PA, USA) via a LabJack A/D converter (LabJack Corp., Lakewood, CO, USA). The input resistance of each filter was measured by the application of 1 mV bipolar pulses of 2-s duration. To eliminate any contribution to the I_sc_ by epithelial sodium channels, 10 μM amiloride was added to the apical chamber. Once the I_sc_ stabilized, roflumilast (100 nM) was included in the apical and basolateral perfusate. After a 20 min pre-treatment with roflumilast, 10 μM albuterol or 10 μM forskolin was added to the apical perfusate, and the resulting increase in chloride conductance was measured as I_sc_. To assure that conductance changes were related to CFTR, I_sc_ decreases upon apical addition of 10 μM CFTR_inh_ 172 were measured as well. To study the combination effect of long acting ß-mimetics and roflumilast, cultures were incubated with 100 nM roflumilast, 100 nM formoterol, both or none for 2 h before exposures to smoke or air. In the Ussing chamber, an additional 10 μM of albuterol was added but to assess the overall influence on CFTR conductance; then I_sc_ decreases upon addition of the CFTR inhibitor CFTR_inh_17 were assessed.

### Airway surface liquid (ASL) volume estimates

ASL volumes from NHBE cells were estimated by meniscus scanning as previously described [20]. Briefly, cultures were washed with phosphate buffered saline (PBS) 24 h prior to measurements to clean the filters from mucus accumulation, which can interfere with ASL reading. At the time of measurement, the 12 mm Transwell supports were placed on a commercially available scanner (Epson perfection V500 PHOTO, Epson America Inc., Long Beach, CA). Scanned menisci were used to estimate ASL volume using software generously provided by Dr. Myerburg (University of Pittsburgh). Calibration of the 12 mm Transwell supports (Costar Corning, Tewksbury, MA, USA) was done by measuring ASL changes after apical addition of different volumes of PBS.

### Bronchoscopic sampling of airway epithelial cells

Bronchial epithelial cells from patients were obtained during bronchoscopy under conscious sedation for other clinical indications as approved by our IRB. After sedation, a cytology brush was passed through the bronchoscope to harvest airway epithelial cells. The brushings were done in the left and right main bronchus just below the carina using eight passages with ten strokes each. After each passage, the brush was withdrawn from the bronchoscope and rinsed in 2 ml of PBS. Cells were collected from current smokers (ten pack year history of smoking), ex-smokers (quit more than 1 year ago) and non-smokers.

### Quantitative PCR

Total RNA was isolated using the Total RNA E.Z.N.A. kit from OMEGA (VWR International, Radnor, PA, USA). The concentration of RNA was determined using the NanoDrop 1000 spectrophotometer (Thermo Scientific, Rockford, IL, USA). Complementary DNA (cDNA) was made from total RNA using the iScript Synthesis Kit (BioRad, Hercules, CA, USA). Messenger RNAs (mRNA) of interest were quantified by PCR using a BioRad iCycler and TaqMan probes (Life Technologies, Grand Island, NY, USA). The difference in the threshold cycle between the targeted gene and GAPDH (ΔCt) was used as the relative level of expression.

### Statistics

Results were evaluated by one-way ANOVA. If a significant difference was found, a parametric (Newman Keuls) or non-parametric analysis (Dunns) using Prism 5 (GraphPad, La Jolla, CA, USA) was used for comparison of groups. *p* < 0.05 was accepted as significant. Error bars in all figures represent SEM.

## Results

### Effect of roflumilast on intracellular cAMP

**Fig. 1:**
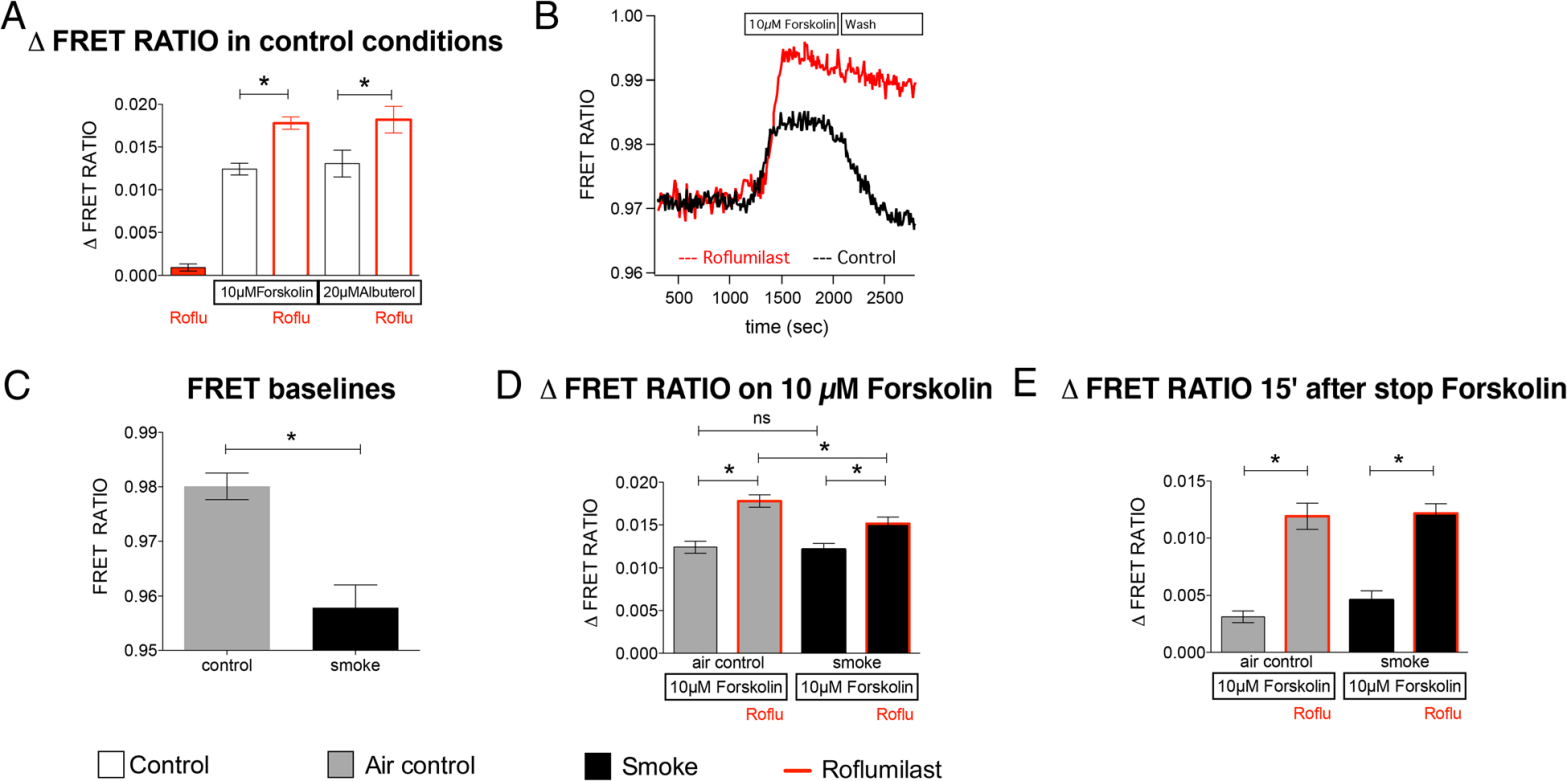
Effect of roflumilast on real-time [cAMP]_i,_ estimated by FRET with and without smoke. **a** Treatment with 100 nM roflumilast alone (10 min) does not increase [cAMP]_i_, but roflumilast enhances forskolin- and albuterol-mediated [cAMP]_i_ increases (10 μM forskolin and 20 μM albuterol in control cultures without airflow exposure (white bars; forskolin three lungs, *n* = 21; albuterol one lung, *n* = 8). **b** Representative tracings of FRET ratios over time as a reflection of changes in [cAMP]_i_ upon stimulation with forskolin in the presence (*red*) and absence (*black*) of roflumilast. **c** Smoke exposed cells (*black bar*) show a decreased baseline FRET ratio compared to cultures exposed to airflow (*grey bar*), possibly indicating a decreased intracellular cAMP level (three different lungs, *n* = 30). However, FRET ratios under these conditions cannot be calibrated to [cAMP]_i_. **d** Roflumilast leads to a significant increase in the forskolin-induced ΔFRET ratio in air control (*gray*) and smoke exposed (*black*) cultures using the Vitrocell VC-10 smoke exposure system (three different lungs, *n* = 21–43). **e** Roflumilast treated cultures show a prolonged elevation of the FRET ratio after washout of forskolin in air control and smoke exposed cells (three different lungs, *n* = 21–43). *p < 0.05

To evaluate the effect of roflumilast on intracellular cAMP concentration ([cAMP]_i_), we used our previously described FRET assay of fluorescently tagged PKA subunits [13] where changes in FRET ratio (ΔFRET ratio) are indicative of changes in [cAMP]_i_ [13]. Apical addition of 10 μM forskolin as well as 20 μM albuterol increased [cAMP]_i_ in NHBE cells. While roflumilast did not increase cAMP when added alone, it increased the peak [cAMP]_i_ response to forskolin and albuterol (Fig. 1a) and prolonged the [cAMP]_i_ increase after forskolin washout (Fig. 1b).

Roflumilast also increased the [cAMP]_i_ peak response and duration to 10 μM forskolin in cultures exposed to smoke or air (air control) in the Vitrocell VC-10 smoking robot. In smoke-exposed cells, the agonist-induced peak ΔFRET ratio response was significantly less compared to the air exposed control cells in the presence of roflumilast (Fig. 1d; each data point from three lungs with *n* ≥ 21 measurements), while no difference was found for the prolonged cAMP increase between smoke and air control (Fig. 1e). To assess the effect of cigarette smoke on intracellular cAMP concentrations, we compared baseline FRET ratios of smoked and air control cultures (Fig. 1c). We found that cultures exposed to cigarette smoke had a significantly lower baseline FRET ratio compared to air control cultures (0.958 ± 0.023 vs 0.980 ± 0.015; 3 lungs, *n* = 30, *p* < 0.05). While calibrations of these baselines are not possible and these results have to take this caveat into account, the data could indicate that acute cigarette smoke exposure decreased intracellular cAMP concentrations. On the other hand, it did not decrease changes in [cAMP]_i_ responses to agonist stimulation and brought FRET ratios in smoked cells to the baseline FRET ratios of control cells in the absence of roflumilast. The further augmenting effect of roflumilast on peak forskolin responses, however, was reduced by cigarette smoke. On the other hand, roflumilast still significantly prolonged the increase in [cAMP]_i_ in smoked cells after forskolin washout.

### Effect of roflumilast on apical chloride conductance

**Fig. 2:**
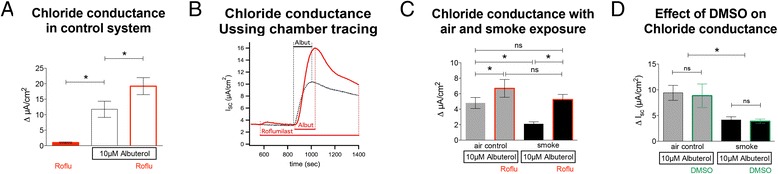
Effect of roflumilast on apical chloride conductance with and without smoke exposure. **a** Evaluation of chloride conductance in control cultures without airflow exposure (*white bars*): Application of roflumilast (100 nM) alone induces a minimal increase in I_sc_ (14 lungs, *n* = 29). Albuterol (10 μM) induces an increase in peak chloride efflux (ΔI_sc_) that is augmented by pre-treatment with roflumilast (100 nM) (six to ten lungs; *n* = 6–10). **b** Representative tracing of an Ussing chamber experiment demonstrates an increase of chloride efflux after administration of 10 μM of albuterol (*black*). Application of roflumilast alone hardly increases the conductance, whereas the addition of albuterol to roflumilast (*red*) treated cultures significantly increases chloride conductance compared to untreated cultures. **c** Chloride efflux is significantly decreased in cultures exposed to cigarette smoke (*black bars*) using the Vitrocell VC-10 smoking robot when compared to air control (*grey bars*). Roflumilast significantly increases apical chloride efflux in air-exposed cells and rescues smoke-associated decreases in chloride conductance (19 lungs; *n* = 19). **d** Control experiments, DMSO (vehicle) does not show any changes in the response of chloride conductance to albuterol in air and cigarette smoke-exposed cultures (two lungs; *n* = 4). *p < 0.05

The effect of roflumilast on apical chloride conductance was measured by short circuit currents (I_sc_) in NHBE cells. Adding 100 nM roflumilast alone (Fig. 2a) resulted in a minimal current change of 0.99 ± 0.22 μA/cm^2^ (14 lungs, *n* = 29), which was significantly less than the changes observed after apical addition of 10 μM albuterol (11.75 ± 2.62 μA/cm^2^, *p* < 0.05). Use of a chloride-free apical solution did not enhance the effect of roflumilast (not shown).

Pretreatment with roflumilast significantly increased the response to albuterol (19.02 ± 2.27 μA/cm^2^; *p* < 0.05). The dynamics of chloride conductance in cultures treated with roflumilast, albuterol and their combination is demonstrated in Fig. 2b. Effects of 100 nM roflumilast on cultures exposed to control airflow or cigarette smoke in the Vitrocell VC-10 smoking robot are shown in Fig. 2c. Roflumilast increased the short circuit current response to albuterol in air-exposed control cells (6.69 ± 1.14 μA/cm^2^ vs 4.8 ± 0.71 μA/cm^2^; *p* < 0.05). Smoke exposure significantly decreased chloride conductance compared to controls (2.1 ± 0.26 μA/cm^2^ vs 4.8 ± 0.71 μA/cm^2^; *p* < 0.05) but roflumilast reversed the smoke effect (5.26 ± 0.65 μA/cm^2^ vs 2.1 ± 0.26 μA/cm^2^; *p* < 0.05) so that changes in I_sc_ in roflumilast-treated, smoked-exposed cultures were similar to untreated airflow controls (5.26 ± 0.65 μA/cm^2^ vs 4.8 ± 0.71 μA/cm^2^; *p* > 0.05). As shown in Fig. 2d, DMSO did have no effect on chloride conductance in air control (9.4 ± 1.5 μA/cm^2^ vs 8.9 ± 2.3 μA/cm^2^) and cigarette smoke exposed cells (4.1 ± 0.6 μA/cm^2^ vs 3.9 ± 0.4 μA/cm^2^: 2 different lungs, *n* = 4).

These data show that roflumilast increased albuterol-stimulated apical chloride efflux and rescued the negative effect of cigarette smoke on this conductance. Addition of roflumilast without additional stimulation with albuterol or forskolin increased I_sc_ only minimally.

### Effect of roflumilast on CFTR function in smoke-exposed cells

**Fig. 3:**
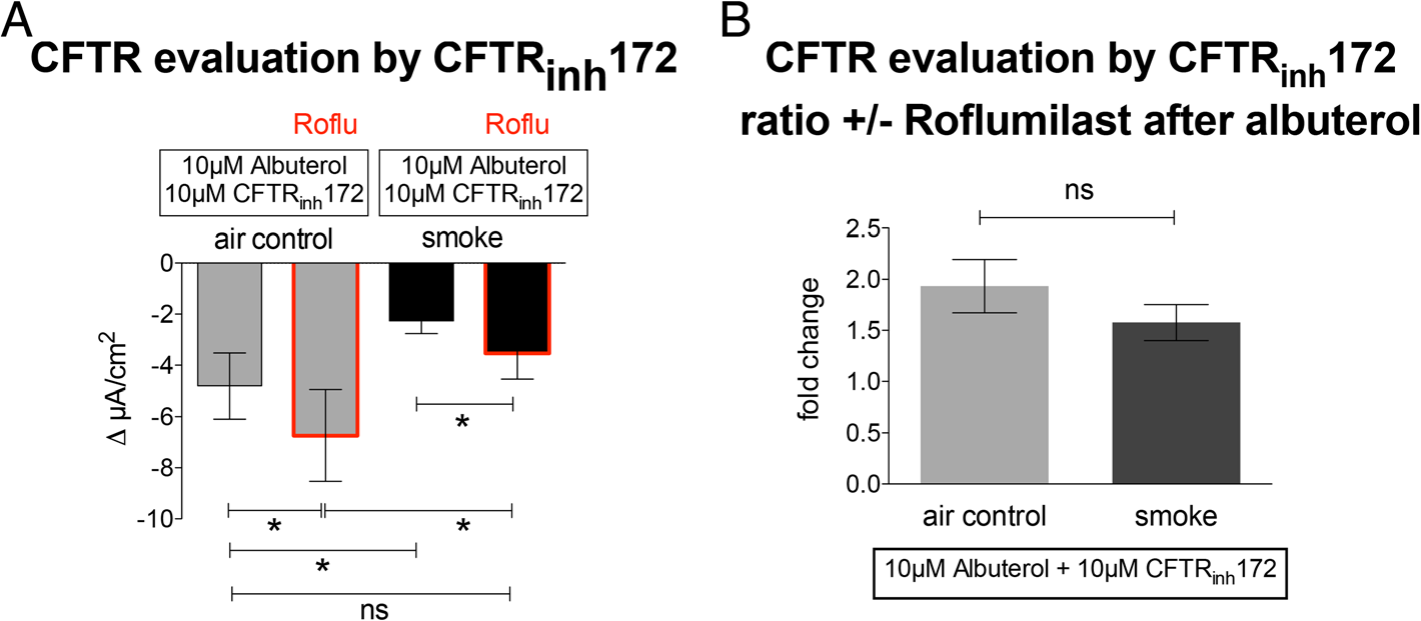
Effect of roflumilast on CFTR function. **a** Basolateral pretreatment of cells exposed to either air flow (air control; *grey bars*) or cigarette smoke (*black bars*) with 100 nM roflumilast improves CFTR function as measured by the decrease in I_sc_ after apical application of 10 μM CFTR_inh_172; all cells were stimulated with 10 μM albuterol prior to CFTR inhibition. Roflumilast rescues CFTR function in smoke exposed cells to a level not different to the untreated air exposed cells. **b** Air and cigarette smoke exposed cultures show similar fold increases in CFTR conductance with and without roflumilast (19 lungs; *n* = 19). * *p* < 0.05

To assure that the observed effects of roflumilast on I_sc_ were truly related to Cl^−^ efflux via CFTR, smoke- or air-exposed ALI cultures were pretreated basolaterally with 100 nM roflumilast, mounted in Ussing chambers, stimulated with 10 μM albuterol and then 10 μM CFTR antagonist CFTR_inh_172 was added apically (Fig. 3a). The observed decrease in I_sc_ is a more specific measure of CFTR activity. Roflumilast increased CFTR-dependent Cl^−^ secretion in air-exposed cells (−6.74 ± 1.79 μA/cm^2^ vs −4.81 ± 1.29 μA/cm^2^; *p* < 0.05, *n* = 19). Smoke exposure decreased CFTR conductance compared to air control (−2.26 ± 0.51 μA/cm^2^ vs −4.51 ± 1.29 μA/cm^2^; *p* < 0.05) and roflumilast improved CFTR function in smoked cells to a level similar to controls (−3.54 ± 1.00 μA/cm^2^ vs −4.51 ± 1.29 μA/cm^2^; *p* < 0.05; Fig. 3a). The ratio of CFTR conductance in response to albuterol in the absence and presence of roflumilast was not statistically different between smoke- and air-exposed control cells (1.34 ± 0.19 vs 1.9 ± 0,37; *p* > 0.05; Fig. 3b). These data show that roflumilast enhanced impaired CFTR function in NHBE cell cultures exposed to cigarette smoke.

### Effect of roflumilast on Airway Surface Liquid (ASL)

**Fig. 4:**
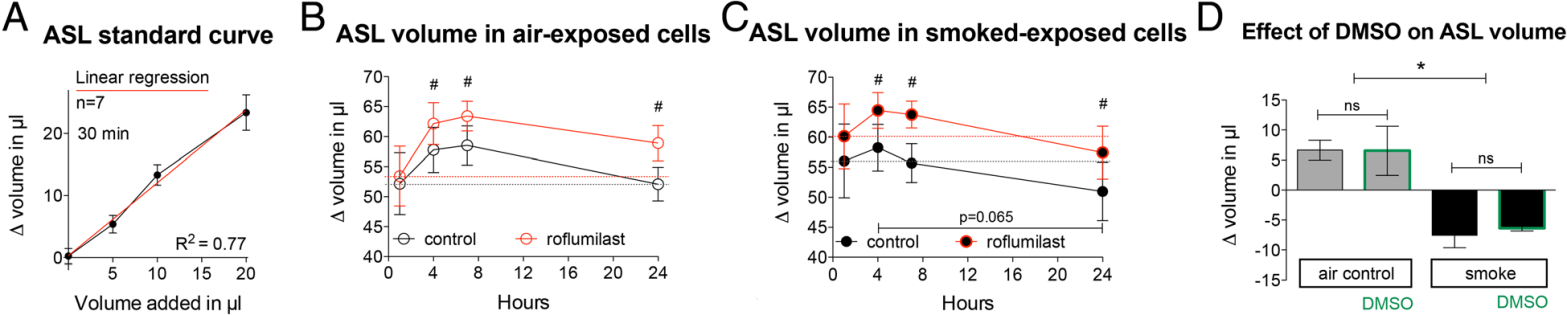
Effect of roflumilast on Airway Surface Liquid (ASL). **a** Calibration of ASL measurements shows a good correlation between added and measured Δ volumes in 12 mm Corning Transwell filters measured 30 min after addition (*n* = 7). Similar results were found when measuring after 3 h (not shown). **b** Following ASL volumes over time after exposure to air shows an initial increase in ASL volume that returns to baseline after 24 h. Roflumilast enhances the response and keeps ASL above baseline at the 24 h time point (six different lungs, *n* = 6, # *p* < 0.05 for comparison between control and roflumilast treated cells at different time points). **c** Following ASL volumes over time after exposure to cigarette smoke shows a blunted initial increase. Roflumilast on the other hand, increases initial volume and restores the ASL volume response after smoke exposure, comparable to air exposure, keeping the ASL volume close to baseline at 24 h (six different lungs, *n* = 6, # *p* < 0.05 for comparison between control and roflumilast treated cells at different time points). Black and red dotted line in B/C for comparison of baseline of control (*black*) and roflumilast treated (*red*) cultures. **d** Control experiments with DMSO as vehicle do not show any difference in Δ ASL measurements between 1 and 4 h in air versus cigarette smoke exposed cultures (two lungs; *n* = 4). * *p* < 0.05

To measure airway surface liquid (ASL) volume, a previously described meniscus scanning method [20] was used as outlined in methods. A good correlation of measured volumes with the meniscus scanning method was found when measuring 30 min and 3 h after addition of 5, 10 and 20 μl PBS to control cells (Fig. 4a). ASL measurements were taken at different time points after smoke or air exposure (Fig. 4b and c). Baseline measurements were taken 1 h after exposure, just prior to roflumilast addition (100 nM; all *n* = 6). Measurements of ASL were done after 4, 7, and 24 h of smoke/air exposure. While air-exposed cells increased ASL over time after exposure to reach baseline again after 24 h, smoke-exposed cells revealed a blunted response with a decrease of ASL that trended to fall below baseline after 24 h (however this did not reach statistical significance). Roflumilast enhanced ASL volume increases in both air- and smoke-exposed cells (Fig. 4b and c). At 24 h, the roflumilast treated, smoke exposed cells had an ASL volume that was significantly higher than the control cells that were only smoke exposed.

These data show that roflumilast enhances ASL volume in NHBE cell cultures and partially reverses the negative effect of cigarette smoke.

### Ciliary Beat Frequency (CBF)

**Fig. 5:**
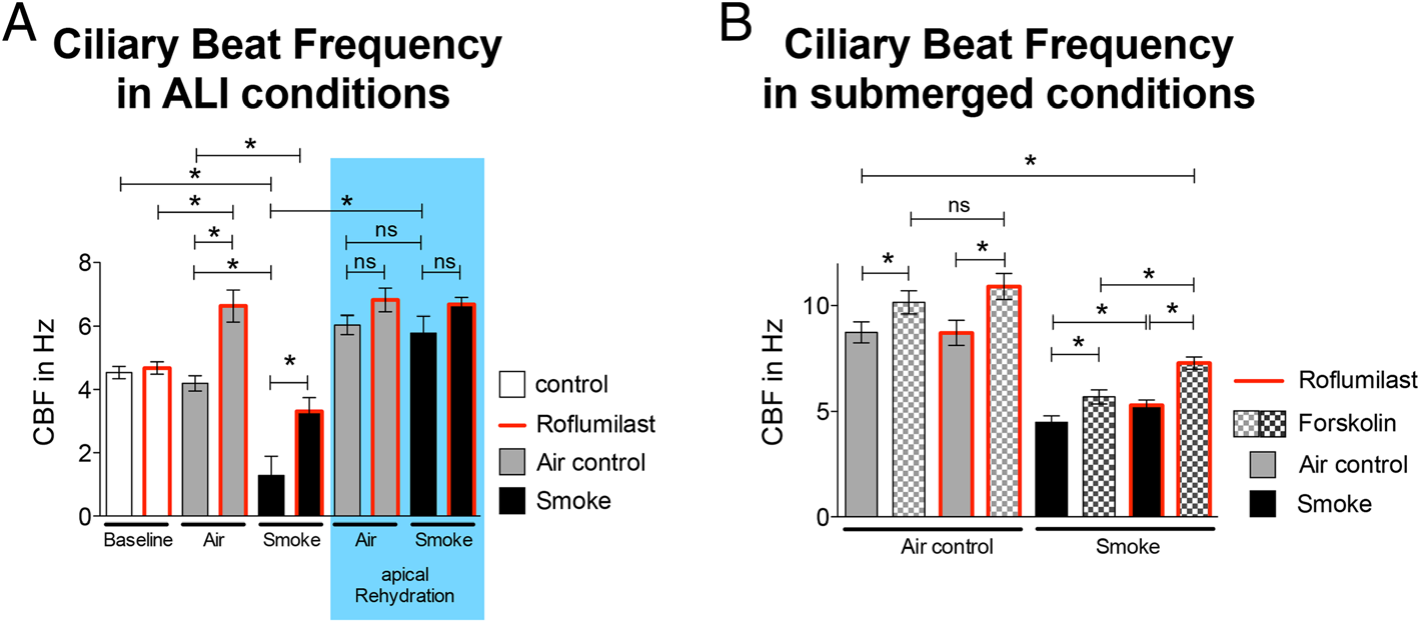
Effect of roflumilast on ciliary beat frequency (CBF). **a** CBF measurement after roflumilast exposure for 4 h (baseline in white bars on the left of the graph after 1 h; five lungs; *n* = 14–40). Roflumilast-treated cultures did not show an increase in CBF compared to untreated cultures (*baseline*). Smoke (4 h after roflumilast) significantly decreases CBF 3 h after exposure, an effect reversed by roflumilast. Exposure to air (air control) increases CBF upon administration of roflumilast (*gray bars*). Bars on a blue background indicate measurements of CBF after rehydration of the apical surface with 50 μl PBS. CBF of cells exposed to smoke vs air equalizes, indicating a role of ASL volume depletion in the CBF changes (* *p* < 0.05). **b** CBF measurements after short-term exposure of roflumilast for 15 min followed by addition of 10 μM forskolin in a submerged, two chamber perfusion system (three lungs; *n* = 10-14). In air control cultures, forskolin increases CBF, but the addition of roflumilast does not further enhance CBF. In smoke-exposed cells, CBF increases upon forskolin addition, and again with roflumilast. CBF baselines are low in smoke-exposed cells, possibly correlating to the lower baseline cAMP levels in smoke exposed cells as assessed by FRET (taken at the same time points). * *p* < 0.05

To examine the effect of roflumilast on smoke-mediated changes in CBF, fully differentiated NHBE cells were used after basolateral treatment with 100 nM roflumilast. DMSO controls were published by us before: DMSO did not show any significant effects on CBF [13]. Baseline measurements were made one hour after roflumilast addition, before the cells were exposed to cigarette smoke or air in the Vitrocell VC-10 robot (control). Three hours after smoke/air exposure (four hours after treatment with roflumilast), CBF was measured. Baseline CBF of the cultures was 4.5 ± 0.2 Hz before and 4.68 ± 0.2 Hz 1 h after exposure to 100 nM roflumilast (*p* > 0.05; five lungs, *n* > 14). Exposure of the cultures to airflow (air control) did not change CBF in untreated cells. However, airflow increased CBF significantly when pre-treated with roflumilast for 4 h (4.19 ± 0.25 Hz vs. 6.63 ± 0.5 Hz; *p* < 0.05). Three hours after exposure to cigarette smoke, CBF decreased compared to air control (1.28 ± 0.06 Hz vs 4.19 ± 0.24 Hz; *n* > 14 each; *p* < 0.05) and non-exposed cultures (4.5 ± 0.2 Hz). Roflumilast increased CBF of the smoke-exposed cells significantly at 3 h (3.31 ± 0.43 Hz vs 1.28 ± 0.06 Hz; *p* < 0.05) and CBF of roflumilast treated, smoke-exposed cultures was not different compared to air-control cultures (3.31 ± 0.43 Hz vs 4.19 ± 0.24 Hz, *p* > 0.05). After initial CBF measurements, 50 μl PBS was added apically to replenish ASL volume. This maneuver equalized CBF in air- and smoke-exposed cells with or without roflumilast (5.78 ± 0.54 Hz vs 6.67 ± 0.22 Hz vs 6.04 ± 0.3 Hz vs 6.82 ± 0.37 Hz; *p* > 0.05 for all comparisons; Fig. 5a), possibly indicating ASL volume loss as the main reason for the CBF decrease rather than a direct smoke effect on cilia. However, a lower [cAMP] after smoke exposure may contribute here as well.

To evaluate the effect of roflumilast on CBF independent of ASL volume, we measured CBF in submerged conditions. Treatment with 10 μM forskolin increased CBF in air control cells in the absence and presence of roflumilast (8.75 ± 0.5 Hz vs 10.15 ± 0.55 Hz and 8.73 ± 0.6 Hz vs 10.9 ± 0.62 Hz respectively; *n* > 10; *p* < 0.05; Fig. 5b). Similar changes were seen in smoke-exposed cells and smoke-exposed cells treated with roflumilast (4.48 ± 0.31 Hz vs 5.69 ± 0.33 Hz and 5.29 ± 0.25 Hz vs 7.29 ± 0.29 Hz respectively; *p* < 0.05). CBF of smoke-exposed cells treated with roflumilast and forskolin had a higher CBF than cells treated only with forskolin (7.29 ± 0.29 Hz vs 5.69 ± 0.33 Hz; *p* < 0.05). This indicates that roflumilast had a stimulatory effect on smoke-exposed cilia directly, independent of ASL volume changes. However, the same phenomenon was not observed in air control cells.

The increased baseline of CBF in these submerged experiments compared to the other CBF measurements after apical PBS addition may be related to the fact that the evaluations were done with constant fluid flow in the apical compartment that may stimulate CBF. These data suggest that the CBF changes in Fig. 5b are not related to ASL volume changes, but rather to an increase in cAMP production (see Fig. 1) directly affecting CBF.

In summary, roflumilast has a dual effect on CBF: it directly reverses smoke-induced CBF decreases and increases smoke-related reductions in ASL volume, thereby indirectly allowing normal ciliary beating. The smoke-induced lower CBF baselines in Fig. 5a and b may be due to the lower baseline cAMP levels after smoke exposure suggested by the FRET experiments discussed above.

### Effects of the long acting ß_2_ adrenergic agonist formoterol with roflumilast

All experiments so far described results using forskolin or the short acting ß_2_ mimetic albuterol to increase cAMP levels. In clinical practice, long acting ß_2_ adrenergic agonists are used for chronic treatment of COPD, whereas short acting ß_2_ agonists are employed as rescue inhalers for symptomatic relief [21]. Based on this fact, we examined the effect of formoterol on CFTR and CBF in the absence and presence of roflumilast in control and smoke exposed cultures.

**Fig. 6:**
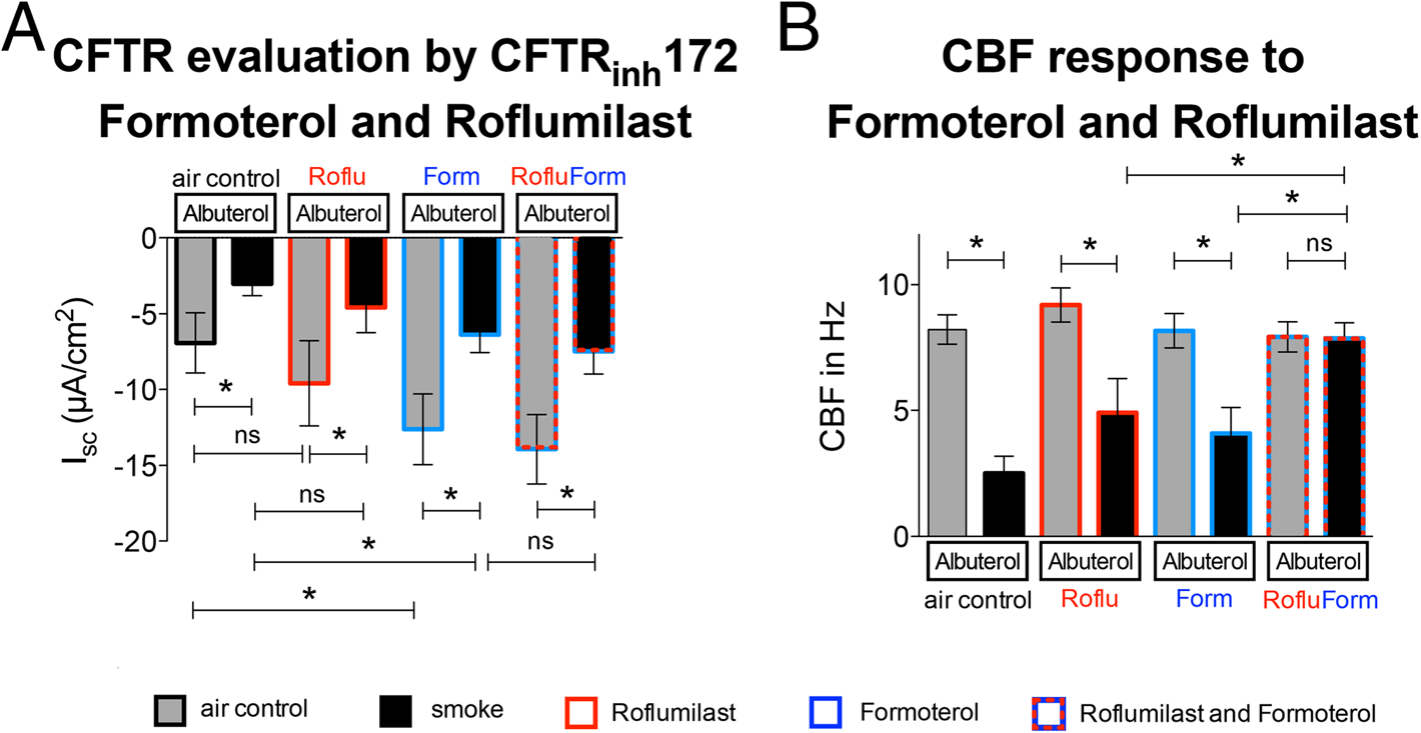
Effect of long acting ß_2_ mimetic in combination with roflumilast on CFTR function and ciliary beat frequency (CBF). **a** CFTR function, assessed by adding 10 μM albuterol followed by CFTR_inh_172, shows a significant decrease in cigarette smoke-exposed cell cultures compared to air control conditions. CFTR function remains significantly lower in smoke exposed cells compared to air controls when adding roflumilast, formoterol or both. Treatment with formoterol improves CFTR function in both smoke and air control compared to non-treated cells, whereas treatment with roflumilast alone under the same conditions does not. **b** CBF is decreased in smoke exposed cells compared to air controls. Neither roflumilast nor formoterol alone rescue smoke-induced decreases in CBF. However, the combination of both restores CBF levels to air control. * for *p* < 0.05, 11 lungs with *n* = 11 for all experiments

Figure 6a shows specific CFTR conductance evaluated by I_sc_ decreases upon channel inhibition with CFTR_inh_172 in the acute presence of 10 μM albuterol in addition to the experimental conditions. Cultures were incubated with 100 mM roflumilast, 100 nM formoterol, both or none for 2 h before adding 10 μM of albuterol. Specific CFTR conductance was significantly decreased in cigarette smoke exposed compared to control cultures (−3 ± 0.79 vs −6.9 ± 1.99 μA/cm^2^). Roflumilast alone did not significantly improve CFTR function either in smoked (−4.58 ± 1.67 μA/cm^2^) or in control cultures (−9.6 ± 2.8 μA). Formoterol alone, however, improved CFTR function (−6.37 ± 1.2 μA/cm^2^ and −12.6 ± 2.3 μA/cm^2^ respectively; 11 lungs, *n* = 11). Next, we evaluated the effect of the combination of formoterol and roflumilast on CBF (Fig. 6b). As expected, CBF was decreased in cigarette smoke exposed versus control cells (2.5 ± 0.7 Hz vs 8.2 ± 0.6 Hz; *n* = 11; *p* < 0.05). Neither roflumilast nor formoterol alone improved CBF. However, the combination of the both significantly increased CBF in smoked cells to a level similar to control cultures (7.9 ± 0.6 vs 7.9 ± 0.6 Hz; 11 lungs, *n* = 11).

### CFTR expression in cells brushed from patients

**Fig. 7:**
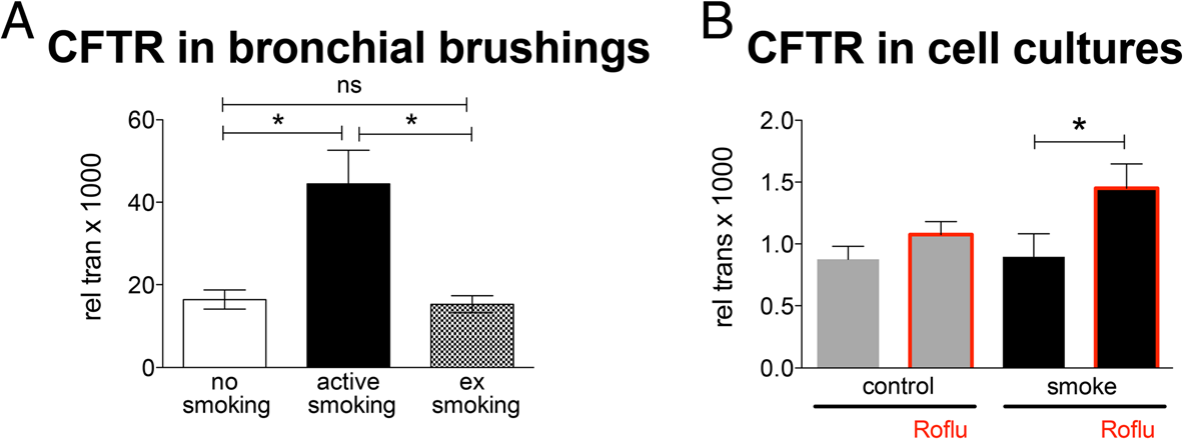
Change in CFTR expression in brushed cells obtained during bronchoscopies and NHBE cultures. **a** Expression of CFTR is increased in brushed cells obtained from airways of smokers compared to non-smokers and ex-smokers. (four to six lungs, *n* = 12–18). **b** In cultured epithelial cells from non-smokers, acute exposure to cigarette smoke does not change CFTR expression compared to control air-exposed cells within 3 h of exposure. Roflumilast (*red framed boxes*) appears to increase CFTR expression in the smoke-exposed cells after 3 h (four lungs, *n* = 12) * for *p* < 0.05

We examined the effect of cigarette smoke and roflumilast on CFTR mRNA expression in airway epithelia cells (Fig. 7). First, qPCR was performed on cells from bronchial brushes obtained during bronchoscopies of individuals with different smoking histories (Fig. 7a). The groups were patients that never smoked, current smokers (at least 10 pack years), and ex-smokers (quit smoking at least 1 year before collection of the cells). We found that current smokers had a significant increase in CFTR expression compared to non-smokers and ex-smokers (44.5 ± 8.1 vs 16.4 ± 2.3 vs 15.3 ± 2.1; expression relative to GAPDH * 1000; each *n* > 12; *p* < 0.05). CFTR expression in ex-smokers returned to the level of non-smokers (*p* > 0.05).

CFTR expression in cultured airway epithelial cells from non-smokers (Fig. 7b) did not increase acutely after exposure to cigarette smoke (four cigarettes) compared to air control (0.89 ± 0.2 vs 0.88 ± 0.1; *p* > 0.05). When air control cultures were treated with 100 nM roflumilast, there was also no significant change in CFTR expression (0.88 ± 0.1 vs 1.08 ± 0.1; *n* = 12; *p* > 0.05). However, when cells from non-smokers, exposed to smoke were treated with roflumilast, an increase in CFTR expression was observed (0.88 ± 0.18 vs 1.45 ± 0.2; *p* < 0.05).

## Discussion

Our results show that inhibition of PDE4 with roflumilast improves parameters of mucociliary clearance in NHBE cells. It increases ASL volume by enhancing apical chloride efflux through CFTR. It also stimulates CBF via an indirect effect on ASL volume and a direct effect on cilia. These changes reverse some of the negative effects of cigarette smoke on MCC and provide further mechanistic evidence that may explain the beneficial effects of this drug on reducing COPD exacerbations.

Following observations that PDE4 inhibition in rodents reduced tobacco smoke-mediated neutrophil influx in BAL, reduced lung parenchyma infiltration of neutrophils, macrophages and lymphocytes, and inhibited endothelial-neutrophil cell interactions [22, 23], initial studies of roflumilast in populations with COPD focused on its anti-inflammatory effects. Roflumilast has been found to reduce sputum neutrophils, eosinophils, and soluble markers of neutrophilic and eosinophilic inflammation compared with placebo [24]. It also decreases other inflammatory molecules such as metalloproteinases and TGF-ß [25–27]. Clinically, this translates not only in a decrease in the rate of mild to moderate exacerbations and a prolongation of the time to the next exacerbation, but also into improved spirometric values and patient-oriented outcomes such as improvements in dyspnea scores [28, 29].

MCC is a key mechanism to protect the airways from inhaled particles and infectious agents. Major components of this apparatus include: 1) effectively beating cilia that move mucus out of the airways and 2) an adequate ASL volume allowing cilia to beat efficiently and hydrating mucus optimally. If MCC fails, patients can develop chronic bronchitis, COPD, bronchiectasis and are prone to pulmonary infections [30]. Our studies may provide some understanding on basic non-inflammatory related mechanisms as to why roflumilast positively influences COPD in patients, namely its effects on ASL volume and ciliary beating.

In our Ussing chamber experiments, 100 nM roflumilast alone did not increase I_sc_ (Fig. 2a). Since phosphodiesterase inhibitors do not directly increase [cAMP]_i_, but decrease its break down, this finding is not surprising, at least for an acute situation. Chronically, it’s possible that roflumilast can elevate cAMP through intrinsic activities of adenylyl cyclases via some basic stimulation by adenosine for instance. This theory is supported by our findings that roflumilast did not change baseline CBF in control cells, but increased it in cells exposed to control airflow (Fig. 5a). Airflow increases ATP release through pannexins [31] and the resulting adenosine increase could stimulate adenylyl cyclase [32], allowing roflumilast to have a significant effect.

In contradiction to our findings, Lambert et al. published a study where the sole addition of 1 nM roflumilast increased I_sc_ close to 10 μA/cm^2^ [33]. Additionally, roflumilast had an EC_50_ of 2.9 nM with a maximal effect of about 40 μA/cm^2^. In order to achieve these high responses with such doses of roflumilast, the authors highlighted the use of a low extracellular chloride concentration (higher driving force). We repeated these experiments but did not observe the I_sc_ stimulations described by Lambert. In addition and in contrast to these authors, we saw a significant effect of 10 μM forskolin addition on I_sc_ after exposure to roflumilast. It is unclear why similar experiments can lead to such different results. One possible explanation is that we used fully differentiated NHBE cells whereas the cited study used primary bronchial epithelial cells grown in submerged monolayers or Calu3 cells. In addition, others have published increases in CBF with sole addition of roflumilast N-oxide [34], an effect only seen upon smoke exposure in our culture system.

Our study showed beneficial effects of roflumilast on NHBE cells exposed to cigarette smoke. Interestingly, smoke did not decrease [cAMP]_i_ responses as measured by FRET, even though it might lower baseline [cAMP], consistent with smoke-induced decreases in CFTR function and ASL volume. Baseline FRET measurements, cannot be calibrated, and therefore these results have to be interpreted with caution. On the other hand, roflumilast rescued decreased apical CFTR conductance and ASL volume by simultaneously increasing [cAMP]_i_. A decrease of cAMP in cigarette smoke extract has previously also been described in bronchial fibroblasts [35].

It has already been known that cigarette smoke reduces CFTR function [7–9] and that this negative effect can be rescued by roflumilast [33]. However, our study goes further by demonstrating that the roflumilast-induced improvement of CFTR function leads to a rescue of ASL volume and a complex improvement of CBF (direct and indirect effect via ASL volume) as well.

The effect of the [cAMP]_i_ on CBF has been well documented in mammals [36–38]. Here we show that CBF increases more than 50 % in smoke-exposed cells in the presence of roflumilast and an additional 25 % after addition of forskolin. Roflumilast also increased CBF by about 50 % in air-exposed cultures. This finding is exciting as the control cultures exposed to airflow represent the airway epithelium in non-smoking individuals simulating air movements during inhalation and exhalation. Furthermore, formoterol, a long acting ß_2_-adrenergic agonist and cornerstone of the treatment of symptomatic COPD [21], enhanced roflumilast’s ability to stimulate CBF in cigarette smoke exposed cells as shown in Fig. 6b.

While alterations in one parameter of mucociliary clearance not always translate into a complementary change of all parameters, it has been shown that a 16 % change in CBF can be associated with a 56 % improvement in mucociliary clearance [39]. Thus, the observed differences with respect to CBF could translate into significant improvements of mucociliary clearance.

Our data contain some unexpected findings. The ASL volume increased initially upon smoke exposure, likely due to mucus secretion. Those initial changes were not necessarily reflected in CBF, indicating that additional mechanisms must be at work that could include surface viscosity. We also showed that airway epithelial cell CFTR mRNA expression is increased in subjects who were active smokers. However, CFTR function and protein expression have been shown to be depressed not only here but also in patients who actively smoke [40]. Thus, mRNA levels of CFTR are not good predictors of the channel’s apical function. Our results show a decreased expression of CFTR in ALI cultures compared to brushes from airways, a finding that has been previously reported [41].

The pathophysiology of COPD is complex. Tobacco smoke induces many anatomical changes including mucus cell hyperplasia and a lower number of ciliated cells with shortened cilia [42] as well as peri-bronchiolar fibrotic changes. Besides its effects on inflammatory cells, PDE-4 inhibition has been shown to decrease EGF-induced MUC5AC expression in human airway epithelial cells [43], to reduce airway mucous metaplasia via its anti-inflammatory properties [44], and to exhibit antifibrotic effects by targeting fibroblasts [45]. Together, these observations along with our findings of additional positive effects on smoke-induced impairment of MCC, provide a more comprehensive mechanistic understanding of the beneficial effects of roflumilast in COPD, in particular for those with chronic bronchitis. While the effect of roflumilast seems clear for improving ciliary function, we do not suggest that this is the only way roflumilast might be beneficial. The effect on ASL volume will affect also mucus hydration and the two effects together will likely have the most significant effect in disease.

## Conclusion

Our results show that roflumilast increases ASL volume and CBF in fully differentiated NHBE cells exposed to smoke by increasing [cAMP]_i_ and in part, by enhancing apical chloride efflux via CFTR.

## Abbreviations

ALI: air liquid interface
ASL: airway surface liquid
BEGM: bronchial epithelial growth medium
CBF: ciliary beat frequency
CFP: cyan fluorescent protein
CFTR: cystic fibrosis transmembrane conductance regulator
FRET: fluorescence resonance energy transfer
KH: Krebs-Henseleit
MCC: mucociliary clearance
NHBE: normal human bronchial epithelium
PBS: phosphate buffered saline
PDE: phosphodiesterase
PKA: phosphokinase A
YFP: yellow fluorescent protein
